# Thermoreversible Diels–Alder Crosslinked Networks in Recycled Poly(ethylene terephthalate) for Reprocessability and Self-Healing

**DOI:** 10.3390/polym18121476

**Published:** 2026-06-12

**Authors:** Yugui Liu, Pengfei Guo, Jianhui Xu, Zengheng Hao, Haidong Liu, Shutong Tang, Junan Shen

**Affiliations:** 1 School of Civil Engineering, Chongqing Jiaotong University, Chongqing 400074, China; liuyugui@cmhk.com; 2 Key Laboratory of Testing Technology for Manufacturing Process, Ministry of Education, Southwest University of Science and Technology, Mianyang 621010, China; kdx1224926@163.com; 3 China Merchants Chongqing Communications Technology Research & Design Institute Co., Ltd., Chongqing 400067, China; haozengheng@cmhk.com (Z.H.); zxtangshutong@cmhk.com (S.T.); 4 National Engineering Research Center for Mountainous Highway, Chongqing 400074, China; 5 Department of Civil Engineering and Construction, Georgia Southern University, Statesboro, GA 30458, USA; shenjunan@hotmail.com

**Keywords:** recycled polyethylene terephthalate, Diels–Alder chemistry, dynamic covalent network, self-healing, reprocessing, polymer upcycling

## Abstract

A thermoreversible dynamic covalent network was constructed in recycled polyethylene terephthalate (RPET) via Diels–Alder (DA) chemistry to enhance mechanical performance, reprocessability, and self-healing. Furan-functionalized RPET (RPET-3F) was first prepared from maleated RPET (RPET-MA), followed by crosslinking with bismaleimide (BMI) at different feed ratios. FTIR spectra confirmed the successful grafting of furan groups and the formation of DA adducts. With increasing BMI content, the gel fraction and crosslink density increased substantially, whereas the swelling ratio decreased, indicating the progressive development of a three-dimensional network. RPET-3F-2B showed the highest network integrity among all samples. DSC analysis revealed a distinct retro-DA dissociation peak at 143 °C and a recrosslinking peak near 124 °C, confirming the thermal reversibility of the DA network. Owing to the optimized network structure, RPET-3F-2B exhibited the best mechanical properties and excellent reprocessability, retaining stable performance after three hot-pressing cycles. After repeated reprocessing, its tensile strength remained 74% higher than that of RPET-MA, while the elongation at break was still improved by about 10%. Moreover, the sample showed efficient thermally induced self-healing at 150 °C, with surface cracks nearly disappearing after 4 h. These results demonstrate that DA chemistry offers a promising route to the high-value reutilization of RPET into recyclable, multifunctional polymer materials.

## 1. Introduction

Poly(ethylene terephthalate) (PET) is a widely used commodity polyester in packaging, fibers, and engineering plastics. Driven by the transition toward a circular economy, converting post-consumer PET into recycled PET (RPET) for high-value applications is of significant importance [1]. However, the practical upcycling of RPET is restricted because repeated thermal processing causes irreversible molecular degradation, which severely limits its performance in demanding engineering applications.

Structurally, PET is a linear semicrystalline polymer whose macroscopic properties depend heavily on molecular weight, chain regularity, and entanglement density. During recycling and melt reprocessing, the combined effects of heat, oxygen, and mechanical shear induce ester bond cleavage. This degradation process reduces molecular weight, broadens molecular weight distribution, and increases carboxyl end-group concentrations, thereby weakening chain entanglements [2]. Consequently, RPET exhibits severe embrittlement, a narrowed processing window, and poor melt stability during subsequent molding or extrusion. Effectively restoring the mechanical integrity of RPET while preserving its thermoplastic processability therefore remains a central challenge in sustainable PET upcycling.

To mitigate the deterioration of RPET, two main strategies have been widely explored: physical toughening and chemical chain extension/branching. Physical modification generally relies on blending RPET with elastomers, inorganic fillers, or nanofillers to improve stiffness and impact resistance [3,4]. Chemical chain extension, in contrast, uses multifunctional reactive molecules to reconnect degraded chain ends, increase molecular weight, and introduce long-chain branching, thereby improving melt strength and mechanical performance [5,6]. Among these approaches, chain extension is often considered one of the most effective routes because it directly addresses the molecular damage caused by reprocessing [7]. To further expand the efficacy of these conventional routes, recent advancements have integrated alternative processing and blending typologies. For instance, reactive extrusion with epoxydic oligomers counteracts thermomechanical degradation by triggering ring-opening reactions with PET terminal groups to stabilize melt rheology [8]. On the other hand, physical blending can be extended to incorporating other polyesters such as poly(butylene terephthalate) (PBT) to regulate crystallization kinetics and minimize thermal warpage [9]. Similarly, promoting in situ transesterification in incompatible systems such as poly(methyl methacrylate) (PMMA) blends serves as an effective pathway to enhance morphological stability [10]. These combined methodologies represent well-established pathways to restore the macroscopic performance of recycled polyesters.

Nevertheless, conventional chain extenders and crosslinkers introduce permanent covalent bonds. Excessive crosslinking enhances strength and dimensional stability but inevitably sacrifices melt reprocessability, failing to reconcile mechanical reinforcement with the recycling needs of thermoplastics.

Dynamic covalent chemistry (DCC) offers a promising strategy to overcome this trade-off. By undergoing reversible bond exchange, dissociation, or reformation at elevated temperatures, dynamic covalent networks impart structural reinforcement during service while preserving material reprocessability [11,12,13,14,15]. For RPET, integrating such dynamic topologies can significantly enhance mechanical modulus, strength, and creep resistance, while efficiently restoring melt flowability under processing conditions. Nevertheless, balancing high-temperature stability with melt processability remains challenging; insufficient network stability compromises mechanical reinforcement, whereas overly stable linkages inevitably hinder flow. To resolve this dilemma, a clear distinction must be made between associative networks (vitrimers), which rearrange via concerted pathways at a constant crosslink density, and dissociative networks based on reversible cleavage. For high-melting-point polyesters like RPET, a dissociative pathway is particularly advantageous, as thermal de-crosslinking temporarily reduces melt viscosity, thereby providing a broadened and efficient processing window.

As a prominent dissociative dynamic reaction, the Diels–Alder (DA) reaction—especially the furan/maleimide pair—is highly attractive due to its catalyst-free, selective, and thermoreversible [16,17]. Typically, DA-based networks can be formed under mild conditions and thermally dissociated via the retro-Diels–Alder (rDA) reaction, enabling self-healing, welding, and reprocessing [18,19,20], even within polyester matrices [21]. Despite these merits, directly applying DA chemistry to RPET remains challenging. On the one hand, the chemical inertness and steric hindrance of the PET backbone limit the efficient grafting of diene or dienophile groups. On the other hand, most reported DA networks rely on solution blending or solvent-assisted multi-step synthesis [18,19,20]. These solvent-dependent protocols increase costs, generate toxic residues or volatile organic compounds (VOCs), and fail to integrate into continuous industrial melt-processing lines, severely hindering the scalable reclamation of large-volume RPET.

An additional challenge stems from the high processing temperature of RPET, where premature rDA dissociation complicates the maintenance of a stable yet reversible network during processing. To address this, indirect functionalization via an anhydride-mediated route offers a viable solution. Anhydride groups react rapidly with amines, efficiently introducing DA-active moieties without compromising the polyester backbone [22]. Compared with direct functionalization, this strategy significantly enhances grafting efficiency and facilitates the subsequent formation of thermoreversible networks directly under solvent-free melt-processing conditions.

Based on this rationale, maleic anhydride-grafted recycled polyester (RPET-MA) was employed as a precursor in this work. Furfurylamine (FAM) was reacted with the anhydride groups to graft furan moieties onto the RPET side chains, yielding the Diels–Alder (DA)-active intermediate RPET-3F. Subsequently, varying molar ratios of bismaleimide (BMI) were introduced to construct a thermoreversible crosslinked network (RPET-3F-xB) via solvent-free melt blending. This reactive extrusion strategy eliminates organic solvents and inherently aligns with existing industrial manufacturing lines, offering strong prospects for scalable processing. The effects of BMI content on the network structure, thermal responsiveness, mechanical profiles, self-healing behavior, and melt reprocessability were systematically investigated. The results demonstrate that the DA/rDA network balances robust mechanical reinforcement with efficient flow recovery during reprocessing, imparting excellent repairability and cyclability to the material. Consequently, this work provides a practical and sustainable route for the high-value upcycling of waste polyester.

## 2. Materials and Methods

### 2.1. Materials

Maleic anhydride-grafted poly(ethylene terephthalate) (RPET-MA, maleic anhydride grafting content: 1.2 wt%) was purchased from Huixin Plastic Chemical Co., Ltd. (Dongguan, China). Furfuryl amine (FAM, >99%), 4,4′-bismaleimidodiphenylmethane (BMI, >96%), toluene (TOL, ≥99.5%), o-dichlorobenzene (DCB, ≥98%), and absolute ethanol (EtOH, ≥99.7%) were obtained from Shanghai Aladdin Biochemical Technology Co., Ltd. (Shanghai, China). All chemicals were used as received without further purification.

### 2.2. Preparation of Materials

#### 2.2.1. Preparation and Purification of Furan-Functionalized RPET (RPET-3F)

Furan-functionalized RPET was prepared via the reaction between the anhydride groups of RPET-MA and the primary amine group of furfuryl amine (FAM). Prior to use, RPET-MA was dried and then melt-mixed in an internal mixer at 150 °C and 50 rpm until a homogeneous melt was obtained. Subsequently, FAM was slowly added at a molar ratio of anhydride groups to amino groups of $n_{MA}:n_{FAM}=1:3$, and the reaction was continued for different time durations under continuous shear.

The crude product was then fully dissolved in toluene at 100 °C under stirring at 30 rpm for 120 min. The resulting solution was precipitated in absolute ethanol and washed repeatedly with ethanol to remove residual unreacted species and other soluble impurities. The collected solid was vacuum-dried at 45 °C for 24 h to obtain the purified furan-functionalized precursor, denoted as RPET-3F.

#### 2.2.2. Construction of Dynamically Crosslinked RPET (RPET-3F-xB)

**Figure 1:**
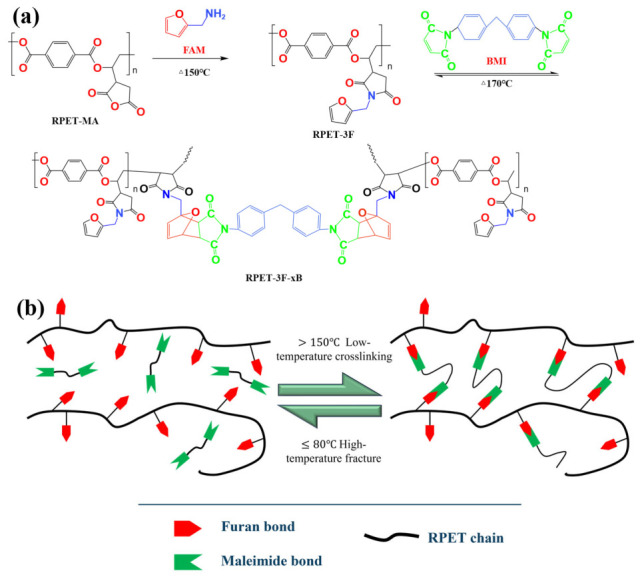
RPET-based dynamic crosslinked networks. (**a**) from furan grafting to Diels–Alder cycloaddition; (**b**) DA/rDA reaction mechanism.

**Figure 2:**
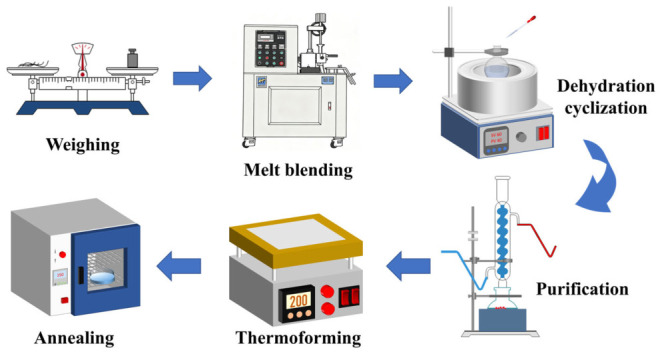
Preparation workflow of crosslinked RPET-3F-xB: From melt-grafting to compression molding.

Purified RPET-3F was introduced into an internal mixer and melt-processed at 170 °C and 60 rpm. BMI was then added according to the predetermined molar ratios of $x=n_{BMI}/n_{MA}=0.5,1,1.5,2$. The blend was further mixed for 30 min to ensure uniform dispersion and to promote the Diels–Alder (DA) reaction between the furan and maleimide groups. After mixing, the material was immediately transferred to a hot press and compression-molded at 200 °C under 10 MPa for 30 min to obtain dense samples. The molded specimens were subsequently annealed in a forced-air oven at 120 °C for 24 h to further drive the forward DA reaction under thermodynamically favorable conditions, thereby promoting the formation of a thermoreversible covalent adaptable network (CAN). The final samples were designated as RPET-3F-xB according to the BMI feeding ratio. The comprehensive preparation strategy and reversible chemistry of the dynamic network are schematically shown in Figure 1. A schematic illustration of the preparation procedure is shown in Figure 2, and the detailed formulations are listed in Table 1.

### 2.3. Characterization and Measurements

The chemical structure, thermal behavior, viscoelastic properties, morphology, and mechanical performance of the pristine matrix (RPET-MA), the furan-functionalized precursor (RPET-3F), and the dynamically crosslinked samples (RPET-3F-xB) were systematically characterized.

#### 2.3.1. Fourier Transform Infrared Spectroscopy (FTIR)

The chemical structures of the samples were analyzed using a Nicolet 6700 spectrometer (Thermo Fisher Scientific, Waltham, MA, USA). Spectra were collected in attenuated total reflectance (ATR) mode within the wavenumber range of 4000–400 ${cm}^{-1}$ at a resolution of $4\;{cm}^{-1}$. FTIR analysis was employed to identify the characteristic functional groups and monitor the chemical structure evolution of RPET-MA, RPET-3F, and RPET-3F-xB.

#### 2.3.2. Solubility and Thermal Reversibility Tests

To evaluate the crosslinking efficiency and network structural integrity, the gel fraction of the samples was determined by solvent extraction. Briefly, dried specimens with an initial mass of $W_{O}$ were immersed in excess o-dichlorobenzene (DCB) and extracted at 150 °C for 24 h. During extraction, the solvent was replaced three times to ensure complete removal of soluble fractions. The remaining insoluble gel was collected, vacuum-dried to constant weight, and weighed as $W_{g}$. The gel fraction ($G_{f}$) was calculated according to:

(1) $$G_{f}=\frac{W_{g}}{w_{o}}\times 100\%$$

The gel fraction reflects the mass ratio of insoluble three-dimensional networks to soluble linear fractions, serving as a primary indicator of crosslinking degree.

Based on the gel fraction results, swelling experiments were further conducted to evaluate the network elasticity and crosslink density of the dynamically crosslinked samples. The crosslink density was calculated using the Flory–Rehner equation:

(2) S=ln(1−VR)+VR+χVR22VS(12VR−VR1/3)

where $V_{R}$ is the volume fraction of polymer in the swollen sample, $V_{S}$ is the molar volume of the solvent, and χ is the polymer–solvent interaction parameter. The value of $V_{R}$ was determined by:

(3) $$V_{R}=\frac{W_{2}}{W_{2}+\left(W_{1}-W_{2}\right)\frac{\rho_{RPET\text{-}3F\text{-}xB}}{\rho_{DCB}}}$$

where $W_{1}$ and $W_{2}$ are the masses of the swollen and dried samples, respectively; $\rho_{RPET\text{-}3F\text{-}2B}$ and $\rho_{DCB}$ are the densities of the polymer network and DCB, respectively. In this study, $V_{S}$ for DCB was taken as 113.3 mL/mol, χ was 0.35 for the DCB/anhydride-modified RPET system, $\rho_{RPET\text{-}3F\text{-}2B}=1.08\;{g/cm}^{3}$, and $\rho_{DCB}=1.30\;g/{cm}^{3}$.

To further investigate the thermoreversible properties of the dynamic network, comparative dissolution experiments were conducted using DCB as the solvent. The samples selected for comparison included Pristine RPET-MA, the furan-functionalized precursor RPET-3F, and the highly crosslinked RPET-3F-2B. In each experiment, 0.5 g of the sample was immersed in 10 mL of DCB and subjected to static heating in an oil bath at 150 °C. Dissolution states were recorded initially (t = 0 h) and after 24 h of heating, followed by cooling to room temperature to monitor gel precipitation or viscosity variations induced by DA linkage re-formation. These visible changes provide qualitative evidence for the thermoreversible dissociation/reconstruction behavior of the dynamic network.

#### 2.3.3. Differential Scanning Calorimetry (DSC)

The thermal transitions of the samples were investigated using a differential scanning calorimeter (DSC Q2000, TA, New Castle, DE, USA). Approximately 5–8 mg of sample was sealed in an aluminum pan and tested under a nitrogen atmosphere with a gas flow rate of 50 mL/min. Heating and cooling scans were performed in the temperature range of 70–200 °C at a rate of 5 °C/min. The endothermic and exothermic events were analyzed to evaluate the dissociation behavior of the retro-Diels–Alder (rDA) reaction and the thermal reversibility of the DA bonds.

#### 2.3.4. Scanning Electron Microscopy (SEM)

The fracture morphologies of the samples were observed using a scanning electron microscope (FE-SEM, JSM-7610F; JEOL Ltd. Tokyo, Japan). The fractured surfaces were sputter-coated with a thin layer of gold prior to imaging. SEM observations were used to analyze the microstructural features of the samples and to correlate phase morphology and fracture characteristics with their mechanical performance.

#### 2.3.5. Mechanical Testing

The tensile properties of RPET-MA, RPET-3F, and RPET-3F-xB samples with different BMI contents were measured using a universal testing machine (UTM, JHY-5000; Xiamen, China) according to ASTM D638 [23]. Dumbbell-shaped specimens were tested at room temperature with a crosshead speed of 500 mm/min. The stress–strain behavior, tensile strength, and elongation at break were analyzed to elucidate the influence of BMI content and dynamic crosslink density on the mechanical performance of the materials.

## 3. Results and Discussion

### 3.1. FTIR Analysis

**Figure 3:**
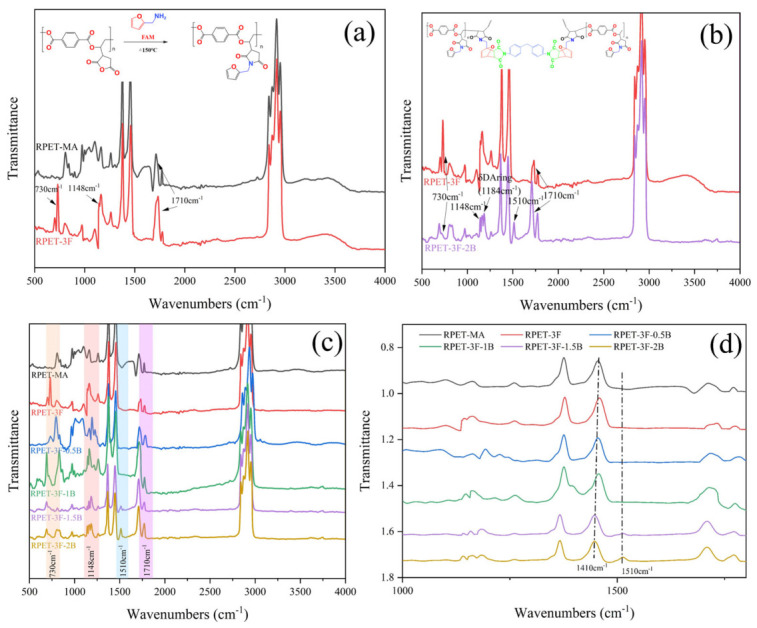
FTIR spectra of the modified RPET networks. (**a**) comparison between RPET-MA and furfurylamine-modified RPET-3F; (**b**) comparison between RPET-3F and the dynamically crosslinked RPET-3F-2B featuring reversible DA bonds; (**c**) spectral evolution of RPET-3F-xB networks prepared with different molar ratios of BMI, including RPET-MA and RPET-3F for reference. (**d**) magnified view of the normalized $1510\;{cm}^{-1}$ peak scaled against the $1410\;{cm}^{-1}$ internal standard.

FTIR spectroscopy was employed to track the chemical structure evolution during RPET-MA furan functionalization and subsequent Diels–Alder (DA) network formation, with the corresponding spectra illustrated in Figure 3.

As shown in Figure 3a, clear spectral changes were observed after the reaction of RPET-MA with FAM. In comparison with RPET-MA, the characteristic carbonyl absorption band around $1710–1718\;{cm}^{-1}$, initially assigned to the anhydride groups, shifted slightly and transformed into imide linkages in RPET-3F. This shift, combined with the emergence of a new absorption band at approximately $1145–1148\;{cm}^{-1}$ (C-O/C-N stretching in imide), confirms that the anhydride groups successfully reacted with the amino groups of FAM to form stable imide structures rather than remaining as maleamic acid intermediates [24].

Furthermore, the introduction of furan moieties was confirmed by the new absorption band at approximately $730–737\;{cm}^{-1}$, attributed to the out-of-plane C–H bending vibration of the furan ring [25,26]. For the DA crosslinked network (RPET-3F-xB), a crucial new band appeared at approximately $1187\;{cm}^{-1}$, representing the coupled C-O and C-N stretching vibrations characteristic of the Diels-Alder adduct [27]. Additionally, the presence of a absorption band at $1513\;{cm}^{-1}$ (aromatic C=C stretching from BMI) further verifies the successful incorporation of the bismaleimide crosslinker and the successful construction of the dynamic covalent network [28]. Importantly, the FT-IR spectrum exhibited a baseline void of characteristic absorption in the $3300–3500\;{cm}^{-1}$ region. The absence of this broad band—typically associated with N–H or O–H stretching vibrations—serves as direct evidence for the complete consumption of reactive precursors and the high purity of the synthesized RPET-3F network.

After the addition of BMI, further structural evolution was observed in the spectra of RPET-3F-xB samples (Figure 3c), providing compelling evidence for the formation of the DA crosslinked network. A new absorption band appeared at around $1510\;{cm}^{-1}$, which is assigned to the skeletal vibration of the aromatic benzene ring in BMI, confirming the successful incorporation of the bismaleimide crosslinker. Crucially, a new characteristic band emerged at approximately 1184–1187 $\;{cm}^{-1}$, attributed to the coupled stretching vibrations of C-O and C-N bonds within the Diels-Alder adducts. The appearance of this peak serves as direct evidence for the covalent crosslinking between the furan-functionalized RPET and maleimide groups [29]. Concurrently, the characteristic carbonyl absorption band near 1710 cm^−1^ exhibited a noticeable increase in intensity compared to RPET-3F, which is ascribed to the additional imide carbonyl groups introduced by BMI [30]. In addition, the intensity of the furan-related band at $730\;{cm}^{-1}$ (out-of-plane C–H bending) significantly decreased, confirming the consumption of furan moieties during the formation of the DA cycloadducts. Furthermore, as clearly illustrated in the normalized and magnified spectra (Figure 3d), the intensity of this $1510\;{cm}^{-1}$ peak scaled against the $1410\;{cm}^{-1}$ internal standard exhibited a monotonic, progressive enhancement from 0.5 B to 2 B, demonstrating a highly controllable incorporation of BMI.

As presented in Figure 3b, the relative intensity of the band at $1184\;{cm}^{-1}$ gradually increased with higher BMI loading, tracking the progressive evaluation of the DA adduct concentration. Consequently, the RPET-3F-2B sample maximizes the dynamic crosslinking density at this optimal stoichiometric ratio, well corroborated by the thorough consumption of the reactive precursors. Overall, the FTIR analysis systematically demonstrates the successful transformation from RPET-MA to a furan-grafted backbone, and finally to a thermoreversible covalent network via DA chemistry.

### 3.2. Solubility Test and Determination of Crosslink Density

**Figure 4:**
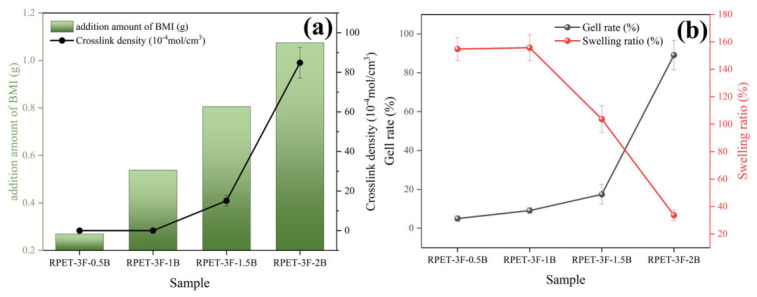
Characterization of the network integrity and crosslinked structure of RPET-3F-xB networks. (**a**) addition amount of BMI and the corresponding crosslink density of different samples; (**b**) evaluation of the gel content and swelling ratio as a function of the crosslink network variation.

The integrity of the three-dimensional network formed through the Diels–Alder (DA) reaction was first evaluated by gel fraction measurements, which directly distinguish the insoluble crosslinked network from soluble linear or branched species. As shown in Figure 4b, the gel fraction remained very low for RPET-3F-0.5B and RPET-3F-1B (below 10%), and only increased slightly for RPET-3F-1.5B (ca. 18%). This result indicates that, at low BMI loadings, the DA reaction mainly generated isolated or loosely connected structures rather than a continuous network, leaving a substantial proportion of soluble chains in the system [30,31]. In contrast, when the BMI content was increased to 2.0 equiv (RPET-3F-2B), the gel fraction rose sharply to nearly 90%, indicating that the system had crossed the gelation threshold and formed a dense, insoluble three-dimensional network. This abrupt increase suggests that BMI content plays a decisive role in transforming the modified RPET from a partially associated structure into a percolated covalent network.

The swelling experiments further confirmed the evolution of the network structure. As shown in Figure 4a, increasing the BMI dosage from 0.269 to 1.075 g led to a dramatic increase in crosslink density from ${0.0114\times 10}^{-4}$ to ${93.5316\times 10}^{-4}\;mol\;{cm}^{-3}$. Meanwhile, the swelling ratio decreased monotonically from 164% to 29% (Figure 4b). Such an inverse correlation between swelling ratio and crosslink density is fully consistent with classical polymer network theory [32]. Specifically, a higher density of DA crosslinking points decreases the network mesh size and restricts chain mobility, thereby enhancing the elastic retractive force to suppress solvent penetration and network expansion [30].

**Figure 5:**
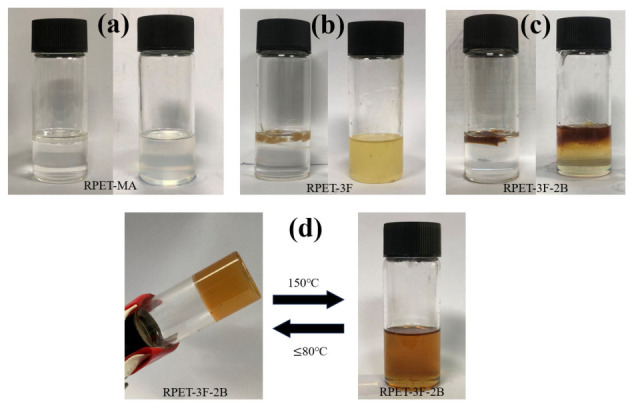
Solubility analysis and thermo-reversibility. (**a**–**c**) Comparative solvent resistance of RPET-MA, RPET-3F, and RPET-3F-2B; (**d**) Thermal-induced de-crosslinking of RPET-3F-2B.

The dissolution states of RPET-MA, RPET-3F, and RPET-3F-2B in DCB, measured initially and after 24 h at 150 °C, are illustrated in Figure 5.

Notably, the most pronounced change occurred between RPET-3F-1.5B and RPET-3F-2B. In this composition range, the gel fraction increased steeply, the crosslink density rose dramatically, and the swelling ratio dropped sharply, indicating that the network structure changed from an incompletely crosslinked state to a highly developed three-dimensional architecture [30,33]. This result also agrees well with the FTIR analysis, which showed that the characteristic bands of the DA adduct became stronger with increasing BMI content. Overall, the results indicate that the crosslinking degree of the RPET system can be effectively controlled by varying the BMI content. Among all the samples tested, RPET-3F-2B exhibited the highest gel fraction, the greatest crosslink density, and the lowest swelling ratio. These findings suggest that RPET-3F-2B forms the most compact and stable thermoreversible covalent network.

### 3.3. DSC Analysis

**Figure 6:**
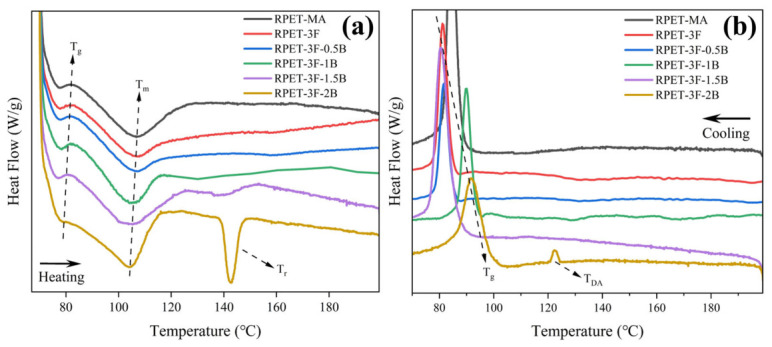
DSC thermograms of the modified RPET materials with varying BMI molar ratios. (**a**) heating scans revealing $T_{g}$ and retro-Diels-Alder endothermic peaks ($T_{r}$); (**b**) cooling scans illustrating $T_{g}$ and the exothermic Diels-Alder re-crosslinking peaks ($T_{DA}$).

Differential scanning calorimetry (DSC) was employed to characterize the heating and cooling behaviors of the samples in order to further verify the formation of the dynamic network in RPET and to evaluate its thermally reversible response [34]. The DSC curves of RPET-MA, RPET-3F and RPET-3F-xB with different BMI contents are shown in Figure 6.

As shown in the heating curves (Figure 6a), all samples exhibited thermal transitions at approximately 80 and 100 °C, corresponding to the glass transition temperature ($T_{g}$) and melting temperature ($T_{m}$) of the RPET-based matrix, respectively [35,36]. More importantly, an additional endothermic peak centered at approximately 140 °C was observed only for RPET-3F-1.5B and RPET-3F-2B. This endothermic event can be assigned to the dissociation of Diels–Alder (DA) adducts via the retro-Diels–Alder reaction ($T_{r}$) [24,25]. The absence of a distinct dissociation peak in RPET-3F-0.5B and RPET-3F-1B suggests that the amount of DA adducts formed at low BMI loadings was insufficient to generate a detectable thermal response [28]. By contrast, RPET-3F-2B exhibited a much stronger and better-defined endothermic peak than RPET-3F-1.5B, indicating the presence of a larger number of thermally reversible DA linkages and, consequently, a higher crosslink density [30]. The cooling curves (Figure 6b) further provide evidence for the thermoreversible nature of the network. Among all samples, only RPET-3F-2B showed a distinct exothermic peak at around 120 °C, which is attributed to the reformation of DA bonds between the furan and maleimide groups during cooling ($T_{DA}$) [24,30]. As the DA reaction is an exothermic cycloaddition process, the appearance of this peak directly confirms the reversibility of the dynamic covalent network [25]. The lack of a discernible exothermic signal in the other samples suggests that the concentration of reversible DA pairs was too low, or that the network structure was not sufficiently developed to produce a clear recrosslinking signal during cooling. These DSC results are highly consistent with the FTIR, gel fraction, swelling, and crosslink density analyses. In particular, RPET-3F-2B showed the clearest retro-DA dissociation upon heating and the most evident DA re-crosslinking upon cooling, demonstrating that this composition possesses the most well-developed thermoreversible covalent network among the investigated samples. Therefore, the DSC analysis further confirms that increasing the BMI content effectively promotes DA network formation, and RPET-3F-2B exhibits the most pronounced dynamic bond-dissociation reconstruction behavior.

### 3.4. Mechanical Properties and Reprocessability

**Figure 7:**
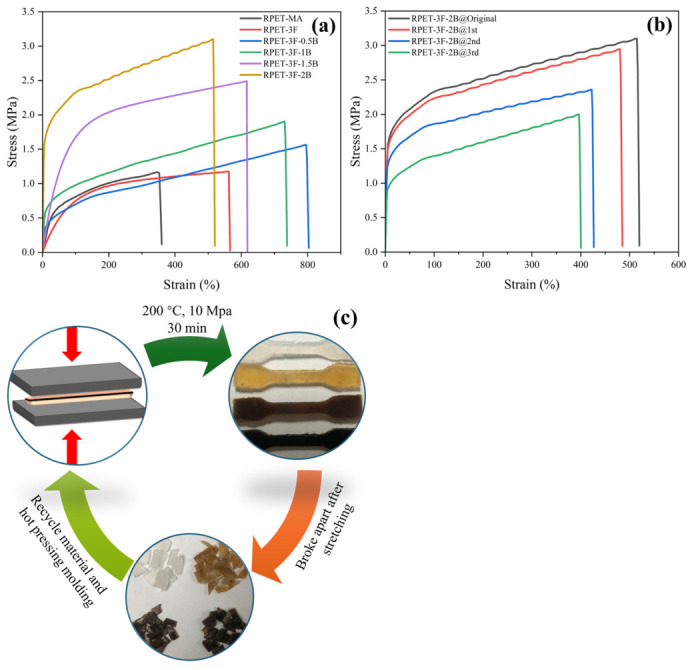
(**a**) Stress–strain behaviors of various RPET samples; (**b**) Mechanical stability of RPET-3F-2B after multiple hot-pressing cycles; (**c**) Workflow of the re-processing procedure.

Figure 7a presents a comparison of the tensile stress–strain responses of RPET-MA, RPET-3F and RPET-3F–xB. Compared to RPET-MA, RPET-3F exhibits higher elongation at break, which can be attributed to the increased free volume and enhanced chain mobility introduced by furan-grafted side groups, thereby improving the matrix toughness [37]. With the addition of BMI, the dynamic DA bonds begin to influence the deformation behavior. At low BMI contents (0.5 B, 1 B), reversible bond exchange reduces local stress concentrations and promotes more cooperative chain motion, leading to simultaneous increases in both stress and strain. When the BMI feed is further raised to 1.5 B and 2 B, the materials exhibit a clear strengthening–detrimental trend: the tensile strength increases to 2.3–3.1 MPa, whereas the elongation at break decreases. This trade-off aligns with polymer network theory, where higher crosslink densities limit chain extensibility and reduce phenomena such as cavitation and segmental pull-out, thereby sacrificing ductility but enhancing load-bearing capacity [36,37]. The compositional dependence is consistent with the findings on crosslink density and swelling behavior presented in Section 3.2, as well as the FTIR data showing the progressive formation of DA-adducts in Section 3.1.

Reprocessability was assessed by hot-pressing and re-cutting the films through repeated “process–recover” cycles (Figure 7c). RPET-3F-2B retained its mechanical performance well over three cycles (Figure 7b). After the first cycle, strength and strain retentions were 95% and 93%, respectively, likely due to minor thermal degradation of the RPET-MA segments and limited loss/rearrangement of crosslink points. The properties then stabilized by the third cycle, indicating that the DA-based network architecture is largely preserved during melt reprocessing [19,38,39]. Notably, even after three cycles, the tensile strength of RPET-3F-2B remains ~74% higher than that of the pristine RPET-MA, while the elongation at break is still ~10% higher. These data demonstrate that the thermoreversible DA network not only enhances the one-time mechanical performance of recycled polyester but also enables efficient multi-cycle reprocessing with minimal property decay.

### 3.5. Self-Healing Behavior

**Figure 8:**
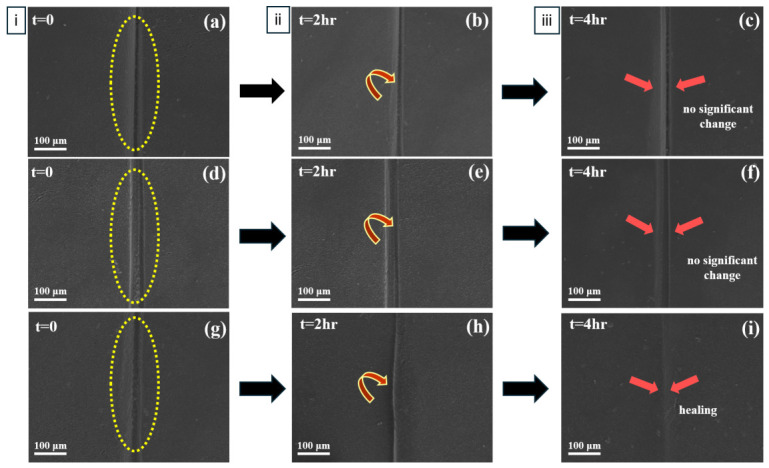
SEM micrographs of surface scratch self-healing behavior at 150 °C for different periods (t = 0, 2 h, and 4 h). (**a**–**c**) RPET-MA, (**d**–**f**) RPET-3F-1.5B, and (**g**–**i**) RPET-3F-2B. Scale bars: 100 μm.

To further evaluate the functionality of the DA-based dynamic network beyond reprocessability, the self-healing behavior of the samples was investigated at the microscopic level by monitoring the evolution of pre-cut surface cracks under thermal treatment. Surface damage was introduced with a blade, and the healing process at 150 °C was followed by SEM, as shown in Figure 8. For RPET-MA, the crack morphology remained nearly unchanged throughout the observation period (Figure 8a–c), indicating that the matrix without a dynamic covalent network could not effectively repair the damaged interface [40,41]. RPET-3F-1.5B exhibited only limited crack closure after heating for 2 h, as evidenced by slight shrinkage of the crack edges, while no obvious further improvement was observed after 4 h (Figure 8d–f). This result suggests that although a certain number of reversible DA linkages had formed in RPET-3F-1.5B, the network was still insufficiently developed to enable efficient interfacial healing. In contrast, RPET-3F-2B showed a much more pronounced healing response. After heating at 150 °C for 2 h, the crack width decreased markedly, and the two fractured edges moved closer together. After 4 h, the crack region had almost disappeared and the surface morphology was restored to a nearly intact state (Figure 8g–i), demonstrating efficient thermally induced self-healing [29,42]. This behavior can be attributed to the reversible dissociation of DA bonds at elevated temperatures, consistent with the DSC results showing the retro-DA process around 140 °C. At 150 °C, partial bond dissociation temporarily relaxes the crosslinked network and increases chain mobility, allowing polymer chains to diffuse across the damaged interface and re-entangle [43]. Upon cooling, the DA bonds reform, thereby reconstructing the network and stabilizing the healed region [44].

These results further confirm that an adequately developed DA network is essential for effective self-healing. Among the investigated samples, RPET-3F-2B exhibited the most efficient crack repair, in good agreement with its higher gel fraction, higher crosslink density, clearer reversible DSC response, and superior reprocessability. Therefore, incorporating an appropriate amount of BMI not only improves the network integrity of RPET, but also endows the material with pronounced thermally triggered self-healing capability.

## 4. Conclusions

In this work, a thermoreversible dynamically crosslinked RPET network was successfully constructed via furan functionalization followed by Diels–Alder (DA) crosslinking with bismaleimide (BMI). FTIR results confirmed the successful grafting of furan groups onto the RPET chains and the subsequent formation of DA adducts after BMI incorporation. With increasing BMI content, the gel fraction and crosslink density increased markedly, while the swelling ratio decreased significantly, indicating the progressive development of a compact three-dimensional network. Among all the investigated samples, RPET-3F-2B exhibited the highest network integrity. DSC analysis further verified the thermoreversible nature of the DA network. A distinct retro-DA dissociation peak was observed during heating. In contrast, a clear DA reformation peak appeared during cooling for RPET-3F-2B, confirming the reversible dissociation/reconstruction behavior of the dynamic covalent bonds. This dynamic network also led to improved mechanical performance. Compared with RPET-MA, the DA-crosslinked samples showed enhanced strength, and RPET-3F-2B achieved the best overall balance between network formation and mechanical reinforcement. More importantly, RPET-3F-2B exhibited excellent reprocessability and self-healing capability. After three hot-pressing cycles, the material still maintained good mechanical performance, with tensile strength remaining 74% higher than that of the original RPET-MA and elongation at break improved by about 10%. SEM observations demonstrated that surface cracks could be effectively healed at 150 °C, which is attributed to the reversible dissociation of DA bonds at elevated temperature and their reformation upon cooling.

Overall, this study presents an effective strategy for imparting thermoreversible dynamic networks to recycled PET using DA chemistry, thereby enhancing its structural stability, reprocessability, and self-healing capabilities. The intact ester backbones and the thermally triggered rDA de-crosslinking characteristics ensure that the modified networks are compatible with classical recycling infrastructures, including both melt re-extrusion and catalytic glycolysis. Consequently, this work provides a promising approach for the high-value and sustainable utilization of recycled polyester materials within existing recycling frameworks.

## Figures and Tables

**Table 1:** Formulations of the RPET-3F-xBMI samples.

Sample Names	Weight of RPET-MA (g)	Weight of FAM (g)	Weight of BMI (g)
RPET-3F	50	1.78	0
RPET-3F-0.5B	50	1.78	0.269
RPET-3F-1B	50	1.78	0.538
RPET-3F-1.5B	50	1.78	0.806
RPET-3F-2B	50	1.78	1.075

## Data Availability

The original contributions presented in this study are included in the article. Further inquiries can be directed to the corresponding authors.
